# Balancing benefit and burden: treatment intensification in paediatric KMT2A rearrangements acute myeloid leukaemia

**DOI:** 10.2340/1651-226X.2025.43878

**Published:** 2025-09-18

**Authors:** Hend Fayez, Mariam Elsherif, Sherine Salem, Nahla Elsharkawy, Amr Elnashar, Mohamed Kamal, Reham Khedr, Leslie Lehmann, Sonia Ahmed, Alaa Elhaddad

**Affiliations:** a Department of Pediatric Oncology, Children’s Cancer Hospital Egypt (CCHE-57357), Cairo, Egypt; b Department of Clinical Pathology, Children’s Cancer Hospital Egypt (CCHE-57357), Cairo, Egypt; c Department of Clinical Pathology, National Cancer Institute, Cairo University, Cairo, Egypt; d Department of Research and Biostatistics, Children’s Cancer Hospital Egypt (CCHE-57357), Cairo, Egypt; e Department of Pediatric Oncology, National Cancer Institute, Cairo University, Cairo, Egypt; f Pediatric Stem Cell Transplantation Unit, Dana-Farber Cancer Institute, Boston, MA, USA

**Keywords:** KMT2A-r, additional cytogenetics, AML, different partners, complex karyotype

## Abstract

**Background and purpose:**

Chromosomal rearrangements involving KMT2A (KMT2A-r) occur in 20% of paediatric acute myeloid leukaemia (AML). Previous studies reported that the outcome depends on the specific fusion partner. The study aimed to report the outcomes of paediatric KMT2A-r AML patients and to assess the impact of different fusion partners.

**Patient/material and methods:**

We retrospectively analysed 610 paediatric patients with intermediate-risk (IR) AML diagnosed at Children’s Cancer Hospital Egypt, from January 2008 to December 2021. Patients were assigned to four groups based on fusion partner.

**Results:**

Of 610 patients diagnosed with IR-AML, 150 (24.6%) had KMT2A rearrangements. KMT2A-r was significantly associated with hyperleukocytosis (*P* = 0.029), central nervous system (CNS) disease (*P* = 0.003), monocytic differentiation (*P* = 0.001), additional cytogenetic abnormalities (ACA) (*P* = 0.04), and complex karyotype (*P* = 0.001). Fusion partner, t(9;11) (p22;q23) (9p22/KMT2A::MLLT3 fusion) was most prevalent (40.8%). KMT2A-r was an independent predictor of relapse with a cumulative incidence of relapse (CIR) of 46% versus 30% in KMT2A negative group (*P* = 0.006). Within the KMT2A-r group, ACA and complex karyotype adversely affected the outcome with 5-year overall survival (OS) of 34% versus 53% (*P* = 0.027) and 26% versus 51% (*P* = 0.004), respectively. Outcome varied depending on fusion partner. Event-free survival (EFS) ranged from 50% to 17%, OS from 54% to 27%, and CIR from 75% to 38%.

**Interpretation:**

KMT2A-r is an independent prognostic factor for relapse, and presence of ACA and a complex karyotype in KMT2A-r patients is associated with poorer outcomes, emphasising the need for aggressive and innovative therapeutic strategies.

## Introduction

Acute myeloid leukaemia (AML) is a genetically heterogeneous disease characterised by a block in differentiation and uncontrolled proliferation of immature myeloblasts in the bone marrow, representing about 20% of childhood leukaemia [1, 2]. Over the recent years, survival has improved, reaching up to 70–80%; this improvement has been achieved in part through refinement of risk stratification based on cytogenetics, molecular abnormalities, treatment responses, as well as advances in supportive care [2]. Chromosomal rearrangements of the KMT2A gene (11q23), seen in 20–25% of paediatric AML patients [3], were classified as one of the recurrent genetic abnormalities in the 22nd edition of the World Health Organization (WHO) [4]. Event-free survival (EFS) for KMT2A rearranged (KMT2A-r) paediatric patients range from 34% to 61% and overall survival (OS) between 44% and 64% [5]. The International Berlin-Frankfurt-Munster Study Group (I-BFM)-SG and other study groups have reported that prognosis is influenced by the KMT2A fusion partner [3]. The most frequently occurring KMT2A-r partner, t(9;11) (p22;q23) (9p22/KMT2A::MLLT3 fusion), is associated with an intermediate prognosis [6]. Also, t(1;11) (q21;q23), (1q21/KMT2A::MLLT11) may be favourable, though this partner is uncommon and thus the impact has not been widely validated [7]. On the contrary, markedly inferior outcomes have been reported for t(4;11)(q21;q23) (4q21/ KMT2A::AFF1 fusion), t(6;11)(q27;q23) (6q27/KMT2A::AFDN fusion), t(10;11) (p12;q23) (10p12/KMT2A::MLLT10 fusion), t(10;11)(p11.2;q23) (10p11.2/KMT2A::ABI1 fusion), and t(11;19)(q23;p13.3) (19p13.3/KMT2A::MLLT1 fusion) (7). In the Children’s Oncology Group (COG)AAML1831(ClinicalTrials.gov identifier: NCT04293562), these five fusion genes have been integrated as unfavourable prognostic indicators into the treatment stratification algorithm [2]. However, other study groups rely only on measurable residual disease assessment (MRD) by multiparametric flow cytometry (MFC) for risk stratification due to the lack of comprehensive international consensus for paediatric KMT2A-r AML [7]. Additional cytogenetic abnormalities (ACAs) have also been identified to be of prognostic value in childhood KMT2A-r AML [7]. They are present in approximately 47% of patients, mainly as chromosomal gains resulting in trisomy 8, 19, 6, and 21 [7]. With the advent of targeted therapies that are fusion partner agnostic, it is important to understand the impact of various KMT2A-r fusion partners on treatment response and outcomes as well as the impact of ACAs and complex abnormalities. Our current study reports on a large number of patients treated at a large paediatric oncology centre, and in addition presents data from a region not typically captured in cooperative group publications.

## Patients/material and methods

### Study design and patients

**Figure 1:**
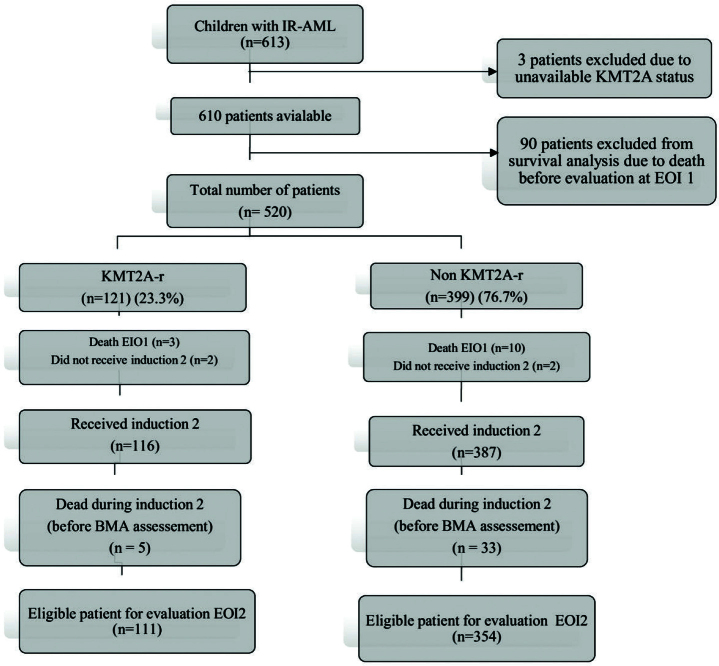
Flow diagram of the cohort of IR-AML children included in the study between January 1, 2008 and December 31, 2021.

We performed a retrospective study of 613 newly diagnosed paediatric AML patients treated at Children Cancer Hospital Egypt 57357 between January 1, 2008, and December 31, 2021 (three patients were excluded from the start and were not included in any analysis because KMT2A status was unavailable in these cases) as shown in Figure 1. A total of 610 patients were eligible, patients were younger than 18 years old, and initially were classified as an intermediate risk (having neither favourable cytogenetics as t(8;21) RUNX1-RUNX1T1, inversion 16 (CBFB-MYH11), nucleophosmin (*NPM1*) or *CEBPA*) nor unfavourable cytogenetics, as monosomy 7, monosomy 5 or *FLT3* Internal Tandem Duplications (*FLT3/ITD*) with high allelic ratio defined as > 0.4 *(FLT3/ITD positive*) (Supplementary Figure 1). Patients with acute promyelocytic leukaemia (APL), Down syndrome AML, therapy-related myeloid neoplasm, Fanconi anaemia, and isolated myeloid sarcoma were excluded from the study. All patients received initial treatment as per the institutional protocol adopted from COG AAML 0531 (ClinicalTrials.gov identifier: NCT00372593) (Supplementary Figure 1) and COG AAML 1031 (clinicaltrials.gov NCT01371981) (detailed treatment shown in Supplement Figure 2). This study was approved by Scientific Medical Advisory Committee (SMAC) and Institutional Review Board (IRB). Patient data were extracted from electronic medical records. Using MFC end induction I (EOI1), patients were restratified into high risk (HR) or low risk (LR) according to the minimal residual disease (MRD) response, with LR defined as MRD < 0.1% and HR with MRD ≥ 0.1 [8]. Patients identified as HR who had a matched sibling donor were offered an allogeneic hematopoietic stem cell transplant (Allo-HSCT) in 1^st^ remission.

From the 610 eligible patients, 90 patients died before evaluation at EOI1, clinical characteristics of those patients are provided in Supplementary Table 4 and survival analysis of the whole cohort including those patients with induction deaths in Supplementary Figure 3.

### Cytogenetic analysis

Bone marrow (BM) samples were cultured and harvested according to standard cytogenetics procedures. Analysis of Giemsa-stained chromosomes was performed according to the International System for Human Cytogenetic Nomenclature. Fluorescence *in situ* hybridisation (FISH) was done using KMT2A break apart probe according to the manufacturer’s instructions (Vysis, Abbott). The slides were analysed using a Leica DM5500 B microscope (Leica Microsystems). Subsequently, images were captured using a JAI video camera and image analyser system (Applied Imaging Ltd). Patients were divided into three distinct fusion partner groups, along with the KMT2A-other group. This KMT2A-other group includes cases of triple-way translocations, unknown KMT2A fusion partners, and other structural abnormalities in the KMT2A gene, such as deletions or partners occurring in fewer than 10 patients, as previously reported by van Weelderen et al. [3]. ACA was defined as any chromosomal aberrations detected by conventional cytogenetic analysis other than KMT2A-r [7], and a complex karyotype was defined as ≥ three cytogenetic abnormalities, whether structural or numerical.

### Multiparametric flow cytometry analysis

MRD assessment was performed for all patients after each cycle of treatment using Multi-parametric Flow Cytometry (MFC) with Navios EX Beckman Coulter, based on the European LeukemiaNet (ELN) proposed consensus using 8–10 colours monoclonal antibody (mAbs) panels. Two methodologies were used, the Leukaemia Associated Immunophenotype (LAIP) approach and the Different from Normal (DFN) approach. MRD was detected based on LAIP’s present on any population and any deviation seen from normal patterns [8].

### Definitions and statistical analysis

Complete remission (CR) was defined as < 5% blasts by morphology and absence of extramedullary disease at EOI1. Refractory disease was defined as ≥ 5% BM blasts morphologically or proven extramedullary disease at end of induction 2 (EOI2) [3]. Relapse was defined as ≥ 5% blasts in the BM or reappearance of blasts in peripheral blood, or the development of extramedullary disease after initial morphologic CR [3]. EOI1 and EOI2 flow-MRD responses < 0.1% were considered negative, and those with MRD ≥ 0.1% considered positive. EFS was calculated from EOI1 to the date of 1st event, events include relapse, refractory disease, death in CR, secondary malignancy, or date of last contact, whichever occurred first [3]. OS was calculated from EOI1 to the date of death or last contact [7]. OS and EFS were calculated using Kaplan-Meier estimates and compared with the log-rank test. The study analysed the baseline characteristics, key outcomes, and other relevant factors using descriptive statistics, including measurements, frequency distributions, and percentages. We compared between groups for categorical variables using the Chi-square test.

Cumulative incidence of relapse (CIR) was defined as the time from EOI1 for patients in CR to relapse, last follow-up, or censoring. The competing event for CIR was death in CR without relapse [7]. The CIR was estimated by adjusting for competing risks and was compared using Gray’s test, which calculates sub-distribution hazard ratios (sHRs). Competing risk regression was performed to estimate hazard ratios (HRs) and 95% confidence intervals (CIs) using R-software (version 4.2). Univariate and multivariate Fine and Gray models were constructed, to identify independent prognostic factors including KMT2A-r status, Total Leucocytic Count, CNS status, complex karyotype, and MRD status at the EOI1 on OS, EFS, and CIR. To validate the findings from the primary competing risks analysis, a cause-specific Cox proportional hazards model was also constructed to specifically assess the hazard of relapse, death without relapse was a censored event. All tests were two-sided, and a *p*-value < 0.05 was considered statistically significant.

## Results

### Clinical characteristics and treatment response of the whole cohort

Out of 610 paediatric IR-AML patients, 150 patients had KMT2A-r (24.6%) (Figure 1). Patients with KMT2A-r tended to be younger, and KMT2A-r was significantly associated with hyperleukocytosis (23.5% vs. 15.5%, *P* = 0.029), central nervous system (CNS) disease (14% vs. 6.3%, *P* = 0.003), and monocytic differentiation FAB M5 (52.7% vs. 10.9%, *P* = 0.001). The incidence of trisomy 8 and complex karyotype was significantly higher among KMT2A-r patients compared to non-KMT2A-r (*P* = 0.032 and 0.001, respectively) (Table 1). In all, 520 eligible patients were evaluable EOI1, 345 (66.3%) attained CR, with no significant differences in CR rates between KMT2A-r and non KMT2A-r patients (69.4% vs. 65.4%, *P* = 0.41). At EOI2, CR rates were increased 93.3% for KMT2A-r group versus 91.5% for non-KMT2A-r group. Only 465 patients had available data on MRD as assessed by MFC, with both groups having comparable rates of achieving MRD negativity at EOI1 (36.7% vs. 29.8%, *P* = 0.17). However, relapse risk was significantly higher among KMT2A-r patients (45.4% vs. 34.8%, *P* = 0.042) (Table 1).

**Table 1:** Clinical characteristics and outcome of IR-AML patients in relation to KMT2A-r.

Variant	Total Cohort *n* = 610 (%)	With KMT2A-r *n* = 150 (24.6%)	Without KMT2A-r *n* = 460 (75.4%)	P. Value
**Age at diagnosis**				
**Median (range)**	4.9 years (0.1–18.8)	3 years (0–16 years)	6 years (0–18)	0.6
**Gender**				0.079
Female	266 (43.6%)	75 (50.0%)	191 (41.5%)	
Male	344 (56.4%)	75 (50.0%)	269 (58.5%)	
Female:male ratio	0.7:1	1:1	0.7:1	
**Initial WBCs**				**0.029**
Median (range)	608	149	459	
(x10^9^)/L)	(0–452)	(0–342)	(0–452)	
< 100	502 (82.6%)	114 (76.5%)	388 (84.5%)	
≥ 100	106 (17.4%)	35 (23.5%)	71 (15.5%)	
Unknown	2	1	1	
**Cytogenetics**				
Normal karyotype	243 (39.8%)	0	243 (52.8%)	
Trisomy (19)	30 (4.9%)	6 (4.0%)	24 (5.2%)	0.934
Trisomy (8)	76 (12.5%)	27 (18%.0)	49 (10.7%)	**0.04**
Trisomy (21)	48 (7.9%)	11 (7.3%)	37 (8.0%)	0.428
Other abnormalities	204 (33.4%)	39 (26%)	165 (35.9%)	**0.04**
Complex karyotype	51 (8.4%)	23 (15.3%)	28 (6.0%)	**0.001**
**CNS involvement**	50 (8.2%)	21 (14.0%)	29 (6.3%)	**0.003**
**FAB classification**				
M0	61 (10.0%)	10 (6.7%)	51 (11.0%)	0.32
M1	84 (13.8%)	9 (6.0%)	75 (16.3%)	**0.002**
M2	119 (19.5%)	6 (4.0%)	113 (24.6%)	**0.001**
M4	99 (16.2%)	31 (20.7%)	65 (14.1%)	0.12
M5	129 (21.1%)	79 (52.7%)	50 (10.9%)	**0.001**
M6	9 (1.5%)	0	9 (2.0%)	
M7	109 (17.9%)	15 (10%)	94 (20.4%)	**0.002**
**Treatment response**				
** EOI1 (*N* = 520)**	520	121	399	0.41
**-BM morphology**	345 (66.3%)	84 (69.4%)	261 (65.4%)	
CR	175 (33.7%)	37 (30.6%)	138 (34.6%)	
No CR				
**-Flow-MRD**	465	109	356	0.173
< 0.1	146 (31.4%)	40 (36.7%)	106 (29.8%)	
≥ 0.1	319 (68.6%)	69 (63.3%)	250 (70.2%)	
Not available	55	12	43	
**Treatment response**				
** EOI2 (*N* = 465)**	444	104	340	0.56
**-BM morphology**	408 (91.9%)	97 (93.3%)	311 (91.5%)	
CR	36 (8.1%)	7 (6.7%)	29 (8.5%)	
No CR	21	7	14	
Not available				
**-Flow-MRD**	362	83	279	0.39
< 0.1	225 (62.2%)	55 (66.3%)	171 (61.2%)	
≥ 0.1	137 (37.8%)	28 (33.7%)	108 (38.8%)	
Not available	103	28	75	
**Relapse (*N* = 194)**	194/520 (37.3%)	55/121 (45.4%)	139/399 (34.8%)	**0.042**
**Allogenic HSCT (*N* = 82)**				
CR1	60	10	50	
CR2	22	6	16	
**Clinical Outcome**				
5-year OS	46.8 – 95% CI (42.4–51.1)	46.1 – 95% CI (36.9–55.3)	46.6 – 95% CI (41.6–51.6)	0.82
5-year EFS	40.2 – 95% CI (35.9–44.5)	39.3 – 95% CI (30.4–48.2)	40.1 – 95% CI (35.2–45)	0.89
5-year CIR	34.0 – 95% CI (29–39)	46.0 – 95% CI (35–56)	30.0 – 95% CI (25–36)	**0.006**

WBC: white blood cell counts; CNS: central nervous system; FAB: French American-British subtype; EOI1: end of induction 1; BM: bone marrow; CR: complete remission; MRD: minimal residual disease; EOI2: end of induction 2; HSCT: hematopoietic stem cell transplantation; OS: overall survival; EFS: event free survival; CIR: cumulative incidence of relapse.

Of the 610 patients, 60 (10%) underwent Allo-HSCT in CR1: 13% of KMT2A-r patients compared to 16.5% of non-KMT2A-r patients.

### Survival analysis for the whole cohort

**Figure 2:**
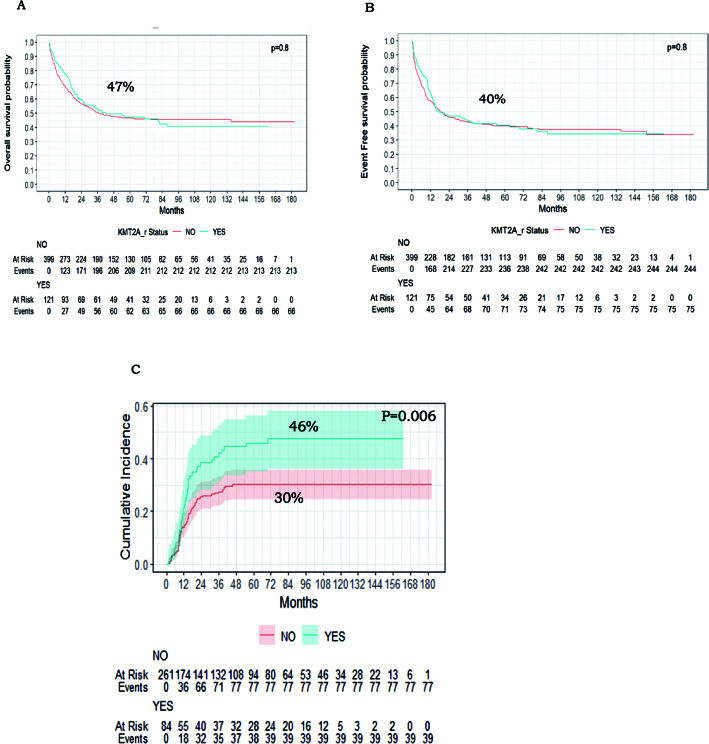
5-year OS, EFS, and CIR curves of patients with KMT2A-r and other IR-AML patients. (A) Kaplan–Meier curves of overall survival of patients with KMT2A-r vs other IR-AML patients. (B) Kaplan–Meier curves of event-free survival of patients with KMT2A-r vs other IR-AML patients. (C) Cumulative incidence of relapse among patients with KMT2A-r vs other IR-AML patients.

There were 520 patients, including 121 with KMT2A-r, who were eligible for survival analysis after excluding 90 patients who died before the evaluation at EOI1. The median follow-up period for the entire cohort was 77.7 months (1.28 to 182.86), a 5-year-OS (47%, 95% CI: 43–51). EFS (40%, 95% CI: 36–45), KMT2A-r did not significantly impact survival (Figure 2A and B). However, KMT2A-r patients experienced significantly higher CIR (46% vs. 30%, HR 1.73, 95% CI: 1.18–2.53) (*P* = 0.006) (Figure 2C). Multivariate analysis using the Fine and Gray model showed that KMT2A-r was an independent predictor of relapse (sHR 1.67; 95% CI: 1.09–2.54; *P* = 0.017) (Supplementary Table 1). This result was confirmed by a complementary multivariate cause-specific Cox model, which also found KMT2A−r to be a significant predictor of relapse hazard (HR, 1.59; 95% CI: 1.04–2.43; *P* = 0.034) (Supplementary Table 1). Although MRD > 0.1 had a notable negative impact on survival, with (HR 0.70; 95% CI: 0.54–0.91; *P* = 0.008) for EFS and (HR 0.71; 95% CI: 0.53–0.94; *P* = 0.018) for OS (Supplementary Table 1).

### Impact of additional cytogenetic abnormality and complex karyotype

**Figure 3:**
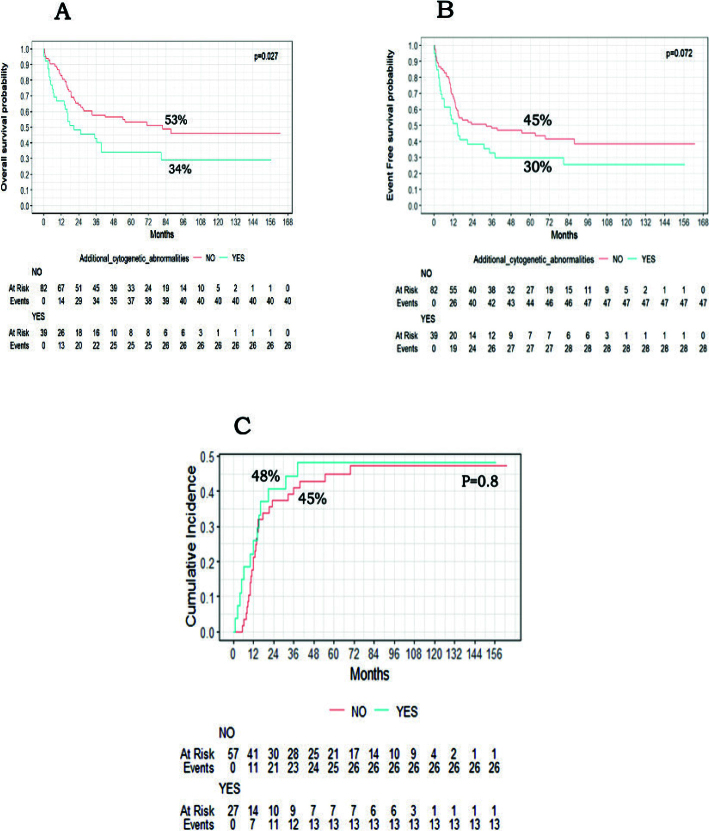
Impact of additional cytogenetic abnormalities on paediatric AML patients with KMT2A-r. (A) Kaplan–Meier curves of overall survival of patients with KMT2A-r and ACA vs other KMT2A-r patients. (B) Kaplan–Meier curves of event-free survival of patients with KMT2A-r and ACA vs other KMT2A-r patients. (C) Cumulative incidence of relapse among patients with KMT2A-r and ACA vs other KMT2A-r patients.

**Figure 4:**
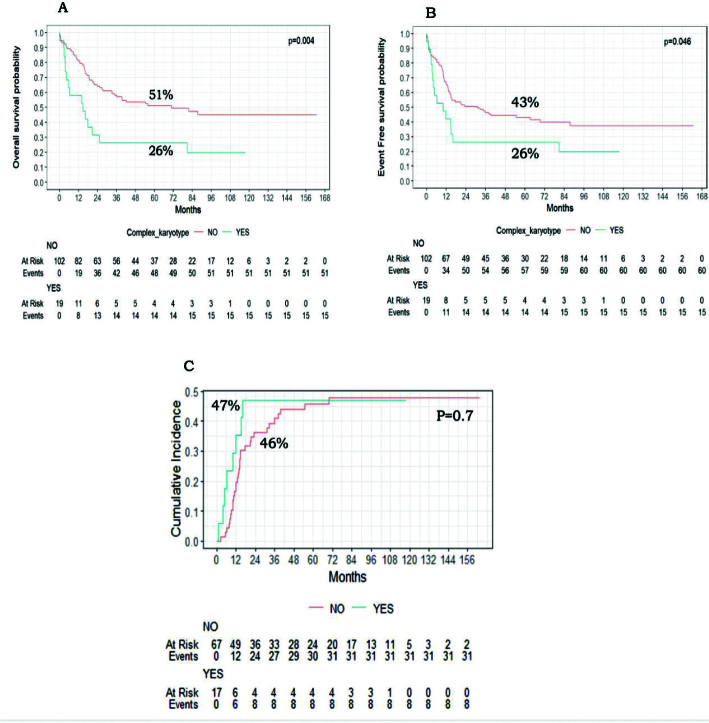
Impact of Complex karyotype on paediatric AML patients with KMT2A-r. (A) Kaplan–Meier curves of event-free survival of patients with KMT2A-r and complex karyotype versus other KMT2A-r patients. (B) Kaplan–Meier curves of overall survival of patients with KMT2A-r and complex karyotype versus other KMT2A-r patients. (C) Cumulative incidence of relapse among patients with KMT2A-r and complex karyotype versus other KMT2A-r patients.

Of the 121 KMT2A-r children, 39 patients (32.2%) had ACA. The presence of ACA adversely affected the outcome with 5-year OS (34% vs. 53%, *P* = 0.027), and 5-year EFS (30% vs. 45%, *P* = 0.072) (Figure 3A and B). Furthermore, the presence of complex Karyotype in 15.7% (*n* = 19/121) of KMT2A-r patients was significantly associated with lower OS (26% vs. 51%, *P* = 0.004) and EFS (26% vs. 43%, *P* = 0.046) (Figure 4A and B). The CIR was not different between patients with ACA or complex karyotype and those without (Figure 3C and 4C respectively).

### Prevalence and outcome of KMT2A-r AML fusion partners

KMT2A-r patients were assigned to three different groups depending on their fusion partner. Partners with less than 10 patients and those with unidentified fusion partners were included in KMT2A-r other group (*n* = 47/121, 38.8%). Among the fusion partners, t(9;11) (p22;q23) (9p22/KMT2A::MLLT3) was the most prevalent representing 40.8% (*n* = 49); conversely t(X;11)Xq24 (KMT2A::SEPT6)( *n* = 3, 2.5%) & t(1;11)1q21/ KMT2A::MLLT11 (*n* = 2, 1.7%) had the lowest incidence (Supplementary Figure 4).

**Figure 5:**
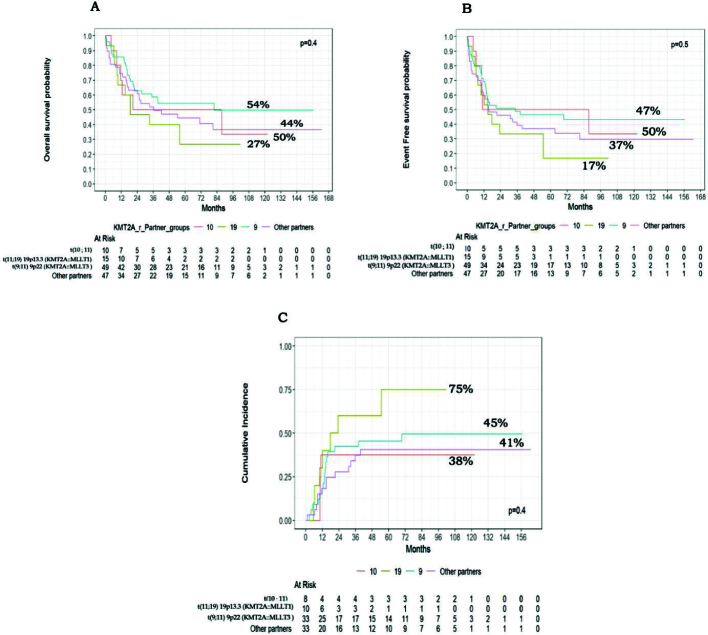
Clinical outcomes of different KMT2A-r partners groups. (A) Kaplan–Meier curves of overall survival of patients with KMT2A-r different partners. (B) Kaplan–Meier curves of event-free survival of patients with KMT2A-r different partners. (C) Cumulative incidence of relapse among patients with KMT2A-r different partners.

Although OS, EFS, and CIR estimates between different KMT2A-r fusion partners did not differ significantly (*P* = 0.4, 0.5, and 0.4, respectively) (Figure 5A–C), lower survival outcomes were observed in patients with t(11;19) 19p13.3 (KMT2A::MLLT1), with 5-year OS, EFS and CIR at 27% and 17%, 75%, respectively (Figure 5). Patients with t(9;11)(p22;q23)(9p22/KMT2A::MLLT3 fusion) had intermediate prognosis with OS, EFS, and CIR at 54%, 47%, and 45% respectively (Figure 5).

Based on fusion partners, KMT2A-r patients were stratified into two groups, the high risk (HR) group (*n* = 30, 24.8%), which included t(11;19) 19p13.3 (KMT2A::MLLT1) (*n* = 15), t(10;11) 10p12 (KMT2A::MLLT10) (*n* = 8), t(6;11) 6q27 (KMT2A::AFDN) (*n* = 5), and t(10;11) 10p11.2 (KMT2A::ABI1) (*n* = 2) and non-HR group (*n* = 91, 75.2%), including t(9;11) 9p22 (KMT2A::MLLT3) (*n* = 49), t(11;17) 17q12 (*n* = 7), t(X;11) Xq24 (KMT2A::SEPT6) (*n* = 3), t(1;11) 1q21/ KMT2A::MLLT11 (*n* = 2) and other partners (*n* = 30).

Clinical characteristics of both groups showed that the HR group had a significantly older age at presentation than the non-HR group, with a mean age of 6.5 years ± 5.1 vs. 4.42 ± 3.9 (*P* = 0.0481), and a higher male predominance (*P* = 0.024). There were no other significant differences in initial disease characteristics (Table 2).

**Table 2:** Outcomes of KMT2A-r AML stratified by fusion-based groups into high risk and non-high-risk groups.

Variant	Total KMT2A-r *n* = 121 (%)	High risk group *n* = 30/121 (24.8%)	Non high risk group *n* = 91/121 (75.2%)	*P*
**Age at diagnosis**				
Means ± SD	2.9 years	6.5 years ± 5.1	4.42 years ± 3.9	**0.0481**
**Gender**				
Female	62 (51.2%)	10 (33.3%)	52 (57.0%)	**0.024**
Male	59 (48.8%)	20 (67.0%)	39 (42.8%)	
Female:male ratio	1.05:1	1:3	1:0.75	
**Initial WBCs**	121	30	91	0.37
(x10^9^)/L)	94 (78.3%)	21(70.0%)	73 (80.2%)	
< 100	27 (21.7%)	9 (30.0%)	18 (19.7%)	
≥ 100				
**CNS involvement**	120	30	90	0.38
Positive	18 (15.0%)	6 (20.0%)	12 (13.3%)	
Negative	102 (85.0%)	24 (80.0%)	78 (86.7%)	
N/A	1	0	1	
**FAB classification**	121	30	91	0.8
M0	12 (10.0%)	0	8 (8.8%)	0.48
M1	7 (5.8%)	1 (3.3%)	6 (6.6%)	0.5
M2	6 (5.0%)	2 (6.7%)	3 (3.3%)	0.62
M4	30 (25%)	10 (33.3%)	19 (20.9%)	0.22
M5	56 (46.3%)	13 (43.3%)	47 (51.6%)	0.75
M6	0	0	0	
M7	10 (8.3%)	2 (6.7%)	8 (8.8%)	0.7
**Cytogenetics**				
(ACA)	39 (32.2%)	7 (23.3%)	32 (35.2%)	0.23
Trisomy (19)	5 (4.1%)	0	5 (5.5%)	0.19
Trisomy (8)	22 (18.2%)	4 (13.3%)	18 (19.8%)	0.43
Trisomy (21)	9 (7.4%)	0	9 (9.9%)	0.073
Other abnormalities	33 (27.3%)	7 (23.3%)	26 (28.6%)	0.57
Complex karyotype	19 (15.7%)	4 (13.3%)	15 (16.4%)	0.68
**Treatment response**				
**EOI1**	121	30	91	0.59
**BM morphology**	84 (69.4%)	22 (73.3%)	62 (68.1%)	
CR	37 (30.6%)	8 (26.6%)	29 (31.9%)	
No CR				
**Flow-MRD**	109	28	81	0.13
< 0.1	40 (36.7%)	7 (25.0%)	33 (40.7%)	
≥ 0.1	69 (63.3%)	21 (75.0%)	48 (59.3%)	
Not available	12	2	10	
**Treatment response**	104	28	76	
**EOI2**	97 (93.3%)	2828 (100%)	69 (90.8%)	0.09
**BM morphology**	7 (6.7%)	0	7 (9.2%)	
CR	7	2	15	
No CR				
**Flow-MRD**	83	23	60	
< 0.1	55 (66.3%)	18 (78.2%)	37 (60.7%)	0.13
≥ 0.1	28 (33.7%)	5 (21.7%)	23 (39.3%)	
Not available	28	7	30	
Relapse	55/121 (45.4%)	17 (56.7%)	38 (41.8%)	0.2
**Allogenic HSCT**	16 (13.0%)	6	10	
CR1	10	5	5	
CR2	6	1	5	
**Clinical Outcome**				
5-year OS	47.3% – 95% CI (38.1–56.4)	32.5% – 95% CI (13.9–51)	51.4% – 95% CI (40.9–61.9)	0.15
5-year EFS	40.5% – 95% CI (31.6–49.4)	30.6% – 95% CI (12.5–48.6)	43.2% – 95% CI (32.9–53.5)	0.33
5-year CIR	46.0% – 95% CI (35–56)	61.0% – 95% CI (35–79)	41.0% – 95% CI (28–53)	0.2

WBC: white blood cell counts; CNS: central nervous system; FAB: French American-British subtype; ACA: Additional cytogenetic abnormalities; EOI1: end of induction 1; BM: bone marrow; CR: complete remission; MRD: minimal residual disease; HSCT: hematopoietic stem cell transplantation; OS: overall survival; EFS: event free survival; CIR: cumulative incidence of relapse.

Notably, HR patients tended to have a lower incidence of achieving MRD negativity at EOI1, at 25% compared to 40.7% in the non-HR group (*P* = 0.13). However, this difference was not reflected in survival outcomes (Supplementary Figure 6A & B). CIR was also not significantly higher in HR patients (61%) versus non-HR group (41%) (*P* = 0.2) (Supplementary Figure 6C).

## Discussion and conclusion

Paediatric AML is a challenging, genetically heterogeneous group of myeloid neoplasms. Over the past few years, survival rates have improved dramatically due to advancements in treatment strategies, the incorporation of targeted therapies, and further refinements in risk stratification [2]. This is particularly important for identifying patients at the highest risk of relapse who may benefit from targeted therapies and/or early HSCT [2]. In cooperative group trials and at our centre, patients with KMT2A-r, one of the most common recurrent cytogenetic abnormalities in paediatric AML, have been identified as intermediate risk and treated with chemotherapy; Allo-HSCT in CR1 is reserved for those with poor MRD response post induction [10].

International collaborative efforts have significantly enhanced our understanding of the prognosis of KMT2A-r (11q23), with over 100 identified fusion gene partners. A study done by the I-BFM revealed that nearly 50% of patients with KMT2A-r failed therapy, even if achieving MRD negativity after the second induction [7]. Additionally, Yuen et al. found that KMT2A-r AML had worse outcomes, with a 5-year EFS of 49%, a 5-year CIR of 38%, and a 5-year OS of 67% compared to paediatric AML patients without KMT2A-r [11].

Our study presents data on a large number of IR AML patients treated with the same approach. We confirm the independent adverse prognostic significance of KMT2A-r on relapse, with a CIR of 46% compared to 30% for non-KMT2A-r patients (*P* = 0.006). However, this did not translate into a difference in OS, with 5-year OS 47% versus 47% (*P* = 0.8) and 5-year EFS 40.0% versus 40.1% (*P* = 0.8).

Previous studies found notable differences in the frequency of various KMT2A-r fusion partners. Balgobind et al. reported KMT2A translocation t(9;11) (p22;q23) (KMT2A::MLLT3 fusion) was the most frequently observed, occurring in 328 out of 756 patients (43%) [9]. These results were supported by the subsequent I-BFM-SG study, which included 1256 children with KMT2A-r AML. The least commonly occurring fusions were t(X;11)(q24;q23) (Xq24/KMT2A::SEPT6 fusion) with 22 cases (1.8%), t(1;11) (p32;q23) (1p32/KMT2A::EPS15 fusion) with 13 cases (1.0%), and t(11;17)(q23;q12) with 10 cases (0.8%) [7]. Our findings, from a geographically distinct population, mirror these results, with the most common identified partner t(9;11) (p22;q23) (KMT2A::MLLT3) (40.4%). In contrast, the least frequently observed partners were t(X;11) (q24;q23) (KMT2A::SEPT6), constituting only 2.5% (3 cases), and t(1;11) (p32;q23) (KMT2A::MLLT11), which accounted for just 1.7% (2 cases).

Pollard et al. and the I-BFM SG demonstrated that the variations in outcomes reported in paediatric KMT2A-r AML was dependent on the fusion gene partner [5, 7]. Patients carrying t(9;11) (q24;q23)KMT2A::SEPT6 and t(1;11) (p32;q23)KMT2A::EPS15 showed favourable outcomes, achieving EFS and OS rates exceeding 75% and 90%, respectively, with a CIR below 20%. Conversely, those with t(10;11) (p11.2;q23) KMT2A::ABI1, t(6;11) (6q27;q23)KMT2A::AFDN, and t(4;11) (q21;q23) KMT2A::AFF1 had significantly poorer outcomes, with EFS rates of just 21.8%, 23.3%, and 25.0%, respectively. Furthermore, patients with t(10;11) (p12;q23) KMT2A::MLLT10 and t(11;19) (p13.3;q23) KMT2A::MLLT1 experienced a high risk of relapse, with EFS rates below 40% [7]. Furthermore, patients with t(10;11) (p12;q23) KMT2A::MLLT10 and t(11;19) (p13.3;q23) KMT2A::MLLT1 experienced a high risk of relapse, with EFS rates below 40% [7]. Our study also showed that t(9;11) (p22;q23) (KMT2A::MLLT3) had an intermediate prognosis, with 5-year OS at 54% and EFS at 47%, similar to the survival outcomes for IR-AML, which had a 5-year OS of 47% and 40% (*P* = 0.3). (Supplementary Figure 5). In contrast, t(11;19) (p13.3;q23) (KMT2A::MLLT1) result in lower survival rates. The 5-year OS, EFS, and CIR were 27%, 17%, and 75%, respectively. Although a low number of different partners prohibited significant differences.

Based on the I-BFM-SG and the ongoing COG AAML study 1831 (NCT04293562), which classify different KMT2A-r partners into high-risk (HR) and non-high-risk (non-HR) groups, our study divided KMT2A-r AML patients accordingly into HR partners comprising 24.8% (*n* = 30), which included t(11;19) (p13.3;q23) (KMT2A::MLLT1), t(10;11) (p12;q23) (KMT2A::MLLT10), t(6;11) (q27;q23) (KMT2A::AFDN), and t(10;11) (p11.2;q23) (KMT2A::ABI1), and the non-HR cohort included 91 out of 121 patients (75.2%), consisting of those with t(9;11) (p22;q23) (KMT2A::MLLT3), t(11;17) (q12;q23), t(X;11) (q24;q23) (KMT2A::SEPT6), t(1;11) (q21;q23) KMT2A::MLLT11, and other partners [5, 7]. The current findings show that approximately 40.7% of the non-HR group reached MRD negativity at EOI1, in contrast to 25.0% of the HR group. The HR group generally showed lower survival rates than the non-HR group, with OS at 33% versus 51% (*P* = 0.2), EFS at 30% compared to 43% (*P* = 0.4), and CIR at 61% versus 41% (*P* = 0.2). Although these differences were not statistically significant, this may be due to the small patient numbers after subgrouping.

Van Weelderen et al. analysed the impact of ACA on KMT2A-r patients and demonstrated worse OS, with OS rates of 56.8% versus 67.9% (*p* < 0.01). However, EFS and CIR were not statistically significantly different [7]. Similarly, our study showed that the presence of ACA among KMT2A-r patients adversely affected survival, resulting in a 5-year OS of 34% versus 54% (*p* = 0.027). It was also associated with lower, though non-significant, EFS at 30% versus 45% for those without ACA. We also examined the impact of complex karyotype and found significantly lower OS (26% vs. 51%, *P* = 0.004) and EFS (26% vs. 43%, *P* = 0.046) compared to those without a complex karyotype.

The limitations of our study stem from its retrospective nature. The inclusion of the flow-MRD response at EOI2 in the entire cohort analysis was hindered because the subgroups became too small due to the presence of multiple KMT2A-r groups. As the use of flow-MRD assays, quantitative polymerase chain reaction, and next-generation sequencing increases, future studies are expected to facilitate accurate detection of MRD in all patients and identify all (cryptic) fusion genes, along with gene mutations, which may further influence outcomes in this disease.

### Conclusion

In conclusion, in a large single-centre study from a geographically under-represented region, we also showed an increased risk of relapse in KMT2A-r AML. These findings contribute to the data that may result in a definitive international consensus on risk group stratification based on different fusion partners and additional findings, allowing patients harbouring KMT2A-r to be classified into intermediate-risk or adverse-risk categories. This will help define the role of allo-HSCT in CR1 independent of treatment response at EOI1. Ongoing discoveries and promising results from new targeted therapeutics like menin inhibitors have shown promising results [12] and may further risk-adapted treatment, ultimately improving the survival of children with KMT2A-r AML.

## Supplementary Material



## Data Availability

The original contributions presented in the study are included in the article/Supplementary Material. Further inquiries can be directed to the corresponding author.
