# Supplementary material for: Balancing benefit and burden: treatment intensification in paediatric KMT2A rearrangements acute myeloid leukaemia
Source: Acta Oncol. 2025 Sep 18;64:43878. doi: 10.2340/1651-226X.2025.43878 (PMC12452033; doi:10.2340/1651-226X.2025.43878)
Supplement: Supplementary file 1 [file AO-64-43878-s1.pdf]

Supplementary material has been published as submitted. It has not been copyedited, or typeset by Acta Oncologica

Supplementary Materials

Supplementary Table 1: Multivariate Analysis of Event-Free Survival, Cumulative Incidence of Relapse, and Overall Survival in whole cohort

Multivariable analysis	OS			EFS			CIR		
	HR	95% CI	P value	HR	95% CI	P value	HR	95% CI	P value
**KMT2A-r Yes/No**	0.95	0.7-1.28	0.724	0.95	0.72-1.26	0.734	1.67	1.09-2.54	**0.017**
**CNS status CNS +VE CNS -VE**	1.52	0.96-2.4	0.072	1.47	0.95-2.26	0.084	1.09	0.52-2.27	0.8
**TLC WBCs < 100 WBCs ≥100**	0.98	0.7-1.37	0.896	0.94	0.68-1.29	0.696	1.05	0.64-1.72	0.9
**Complex karyotype Yes/No**	11.34	0.86-2.08	0.196	1.3	0.85-1.98	0.230	0.8	0.38-1.67	0.6
**MRD EOI1 <0.1 ≥ 0.1**	0.71	0.53-0.94	**0.018**	0.7	0.54-0.91	**0.008**	1.06	0.71-1.57	0.8

HR=Hazard Ratio,CI=Confidence interval,EFS: Event Free Survival, OS: Overall Survival, CIR: Cumulative incidence of relapse

Supplementary Table 2: Outcomes of 121 children with KMT2A-r AML and stratified by fusion-based partners

MLL partners	t (10;11) n=10	t (11;19) n=15	t (9;11) n=49	KMT2A-r other group n=47
5-year OS*	50%	27%	54%	44%
5-year CIR**	38%	75%	45%	41%
5-year EFS***	50%	17%	47%	37%

* OS: Overall Survival.

** CIR: Cumulative incidence of relapse

***EFS: Event Free Survival

Supplementary Table 3: Distribution of additional cytogenetic abnormalities and complex karyotype among different KMT2A-r partners

KMT2A-r partners (N= 121)	With ACA (N= 39)	With complex karyotype (N= 19)
t(9;11) 9p22 (KMT2A::MLLT3) (n=49)	**18 (46%)**	**7 (36.8%)**
t(11; 19)19p13.3 (KMT2A::MLLT1) (n=15)	4 (10%)	2 (10.5%)
t(10 ;11) (n=10)	2 (5%)	1 (5%)
t(11;17) 17q12 (n=7)	0	0
t(6 ;11) 6q27 (KMT2A::AFDN) (n=5)	1 (2.5%)	1 (5%)
t(x ;11) Xq24 (KMT2A::SEPT6) (n=3)	0	0
t(1 ;11) 1p32 (KMT2A::EPS15) (n=2)	1 (2.5%)	1 (5%)
Other partners (n=30)	13 (33.3%)	7 (36.8%)

*Abbreviations, ACA (Additional cytogenetic abnormalities)

Supplementary Table 4 : Clinical Characteristics of 90 patients excluded from survival analysis

Variant	Total Cohort n= 90 (%)
**Initial WBCs (x109)/L)**<br><100<br>≥ 100	 70<br>20
**CNS involvement**<br>Positive<br>Negative<br>Not available	 12<br>64<br>14
**KMT2A-r status**<br>KMT2A +VE<br>KMT2A -VE	 29<br>61
**Started treatment**<br>Yes<br>No	 84<br>6
**Causes of death**<br>Due to disease<br>Covid pandemic<br>Sepsis	 35<br>6<br>32

*Abbreviations, WBCs (white blood cell counts), CNS (central nervous system), EOI 1(End of induction 1)

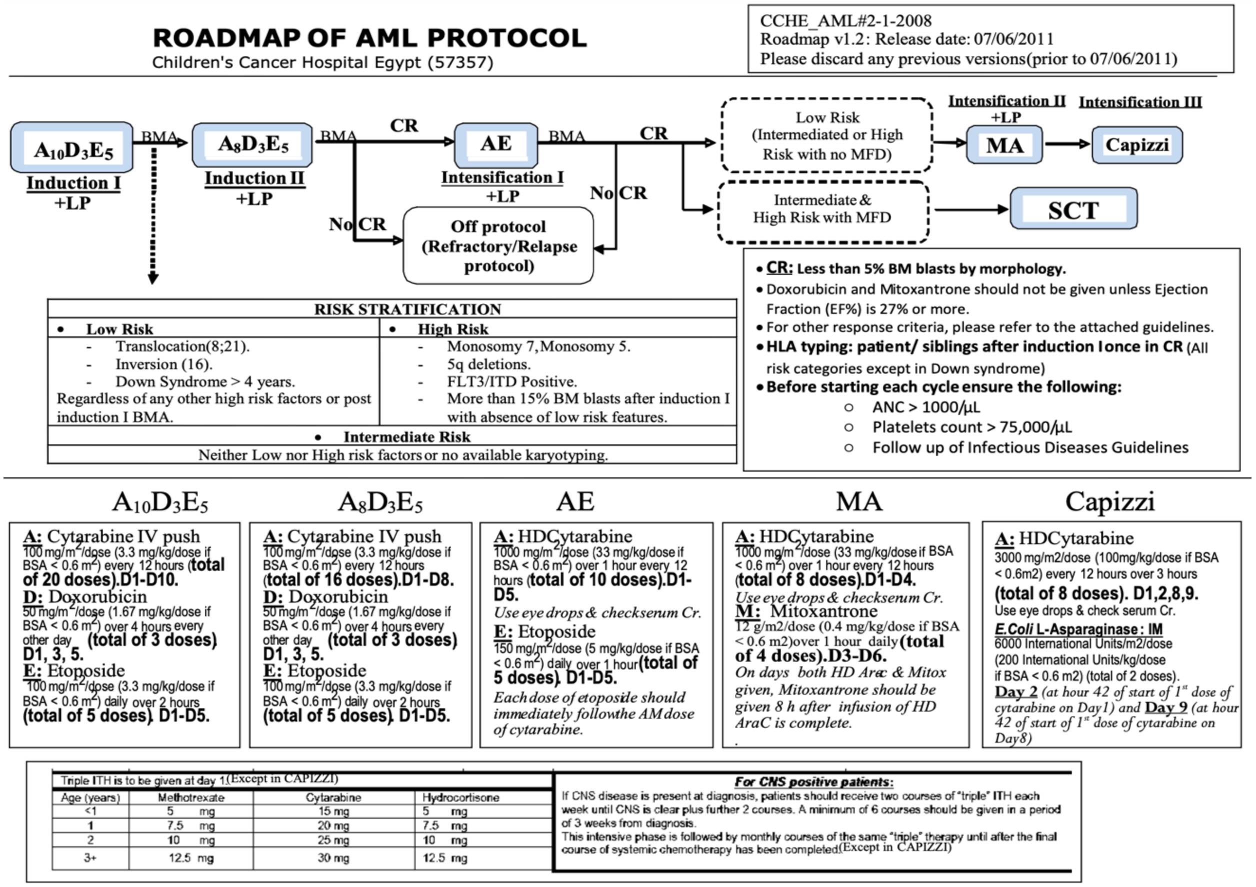

Supplementary Figure 1: Treatment Protocol Roadmap adopted from modified COG AAML 0531

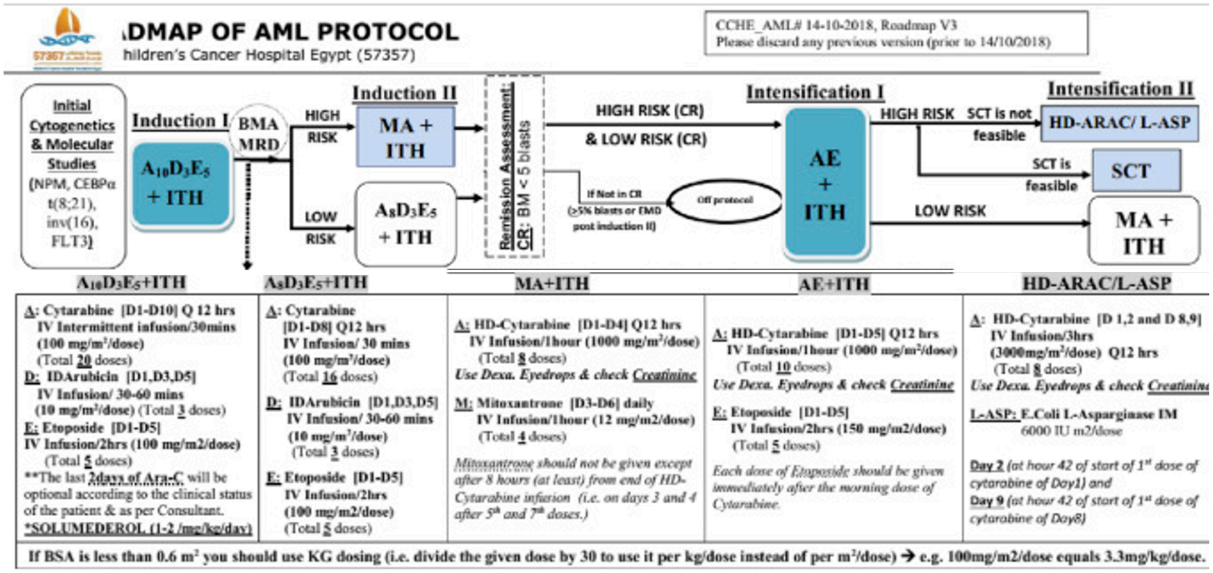

Supplementary Figure 2: Treatment Protocol Roadmap adopted from modified COG AAML 1031

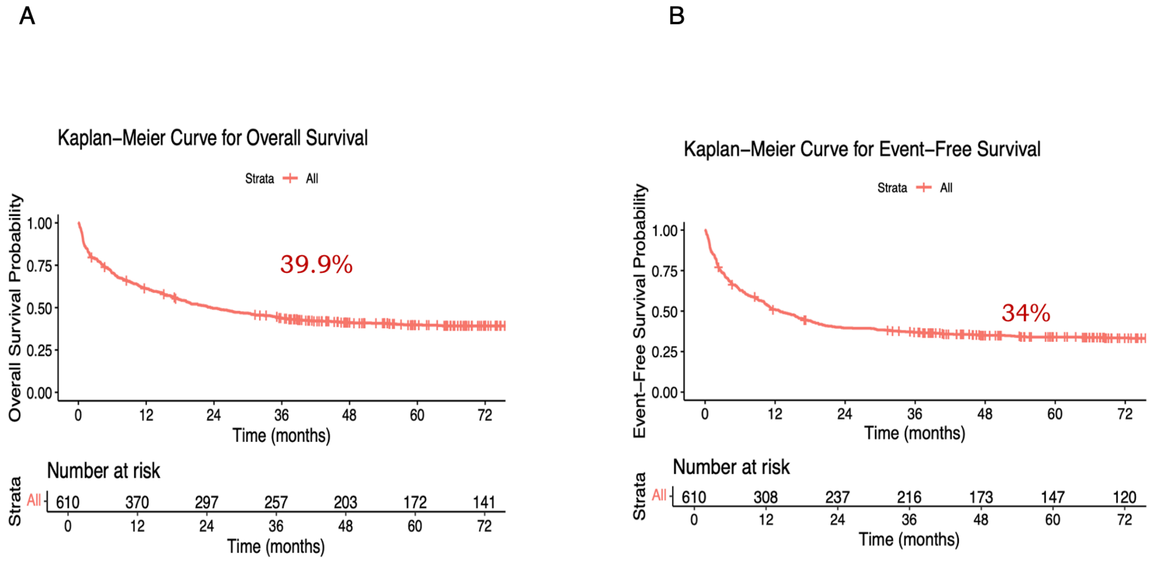

Supplementary Figure 3: Outcomes of whole cohort including 90 patients with induction deaths. (A) OS (Overall survival), **(B)** EFS (Event free survival).

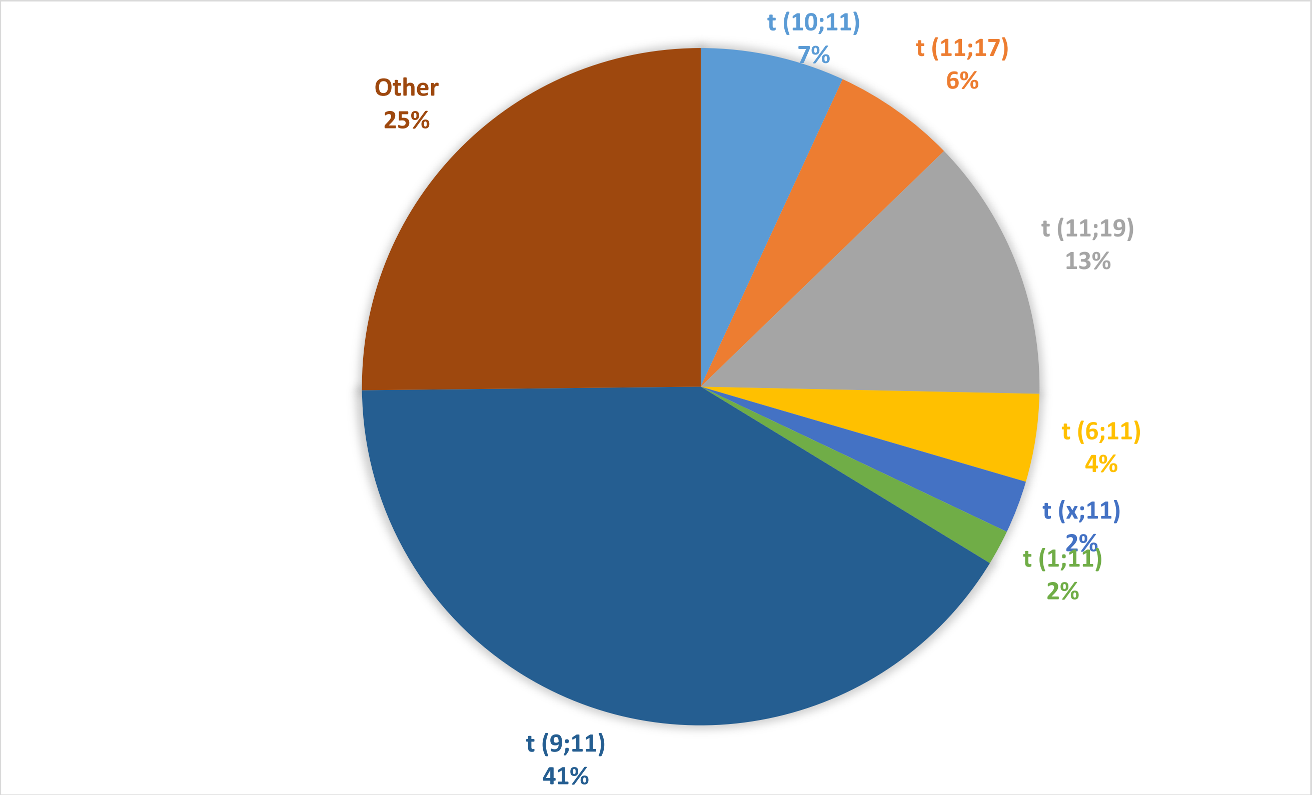

Supplementary Figure 4: Distribution of the fusion based KMT2A-r partners in our cohort of childhood KMT2A-r IR-AML. t(9;11)(p22;q23) (9p22/KMT2A::MLLT3) (n=49) , t(11;19) 19p13.3 (KMT2A::MLLT1) (n=15), t(10;11) (n=10), t(11;17) 17q12 (n=7), t(6;11) 6q27 (KMT2A::AFDN) (n=5), t(X;11) Xq24 (KMT2A::SEPT6) (n= 3) & t(1;11) 1q21/ KMT2A::MLLT11(n= 2). There were 30 were included in other groups including structural abnormalities in KMT2A gene, triple translocations & other patients with unknown partners.

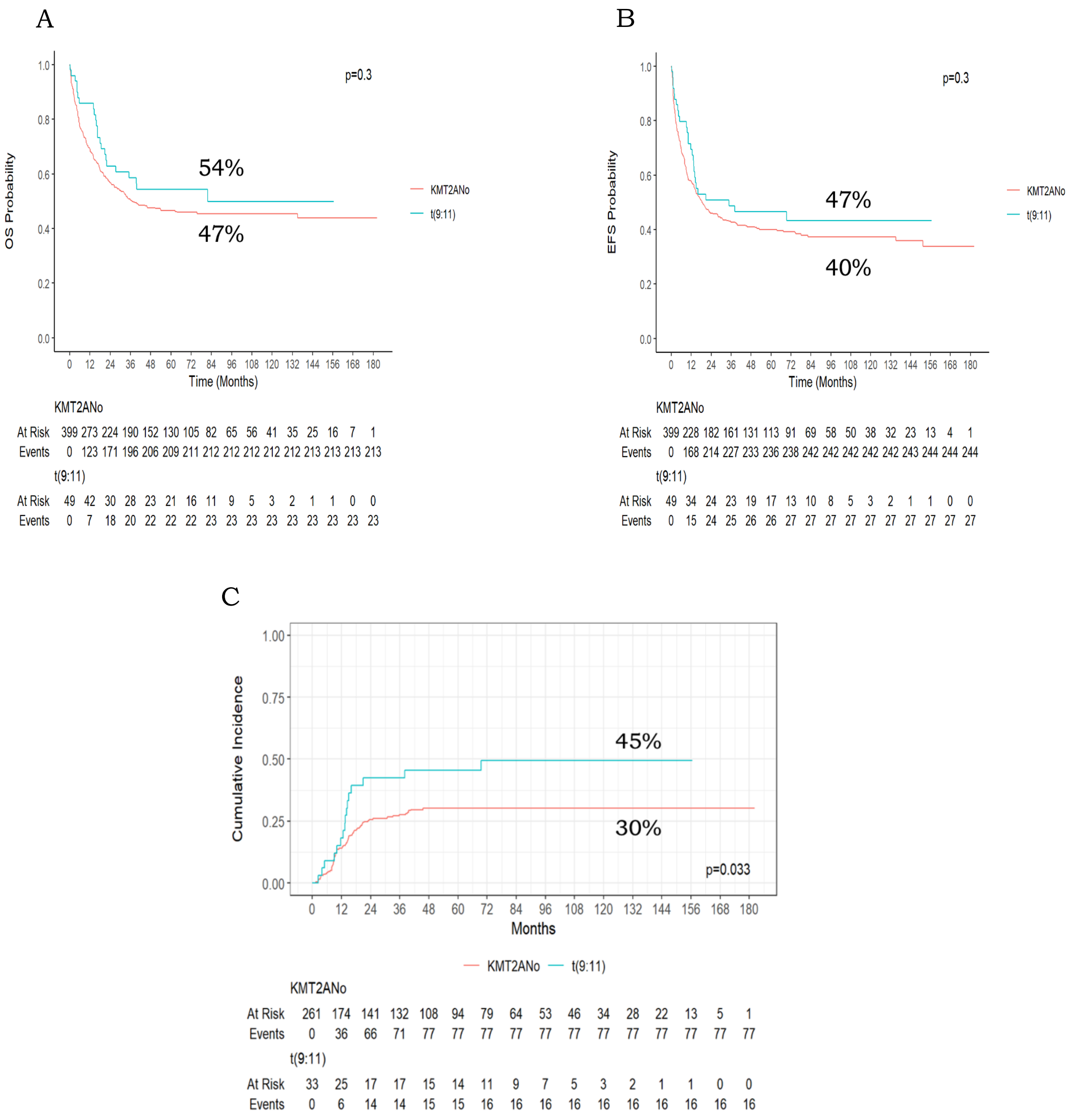

Supplementary Figure 5: Outcomes of t(9;11) in comparison to other IR-AML patients with KMT2A-no. (A) OS (Overall survival), **(B)** EFS (Event free survival), **(C)** CIR (Cumulative incidence of relapse).

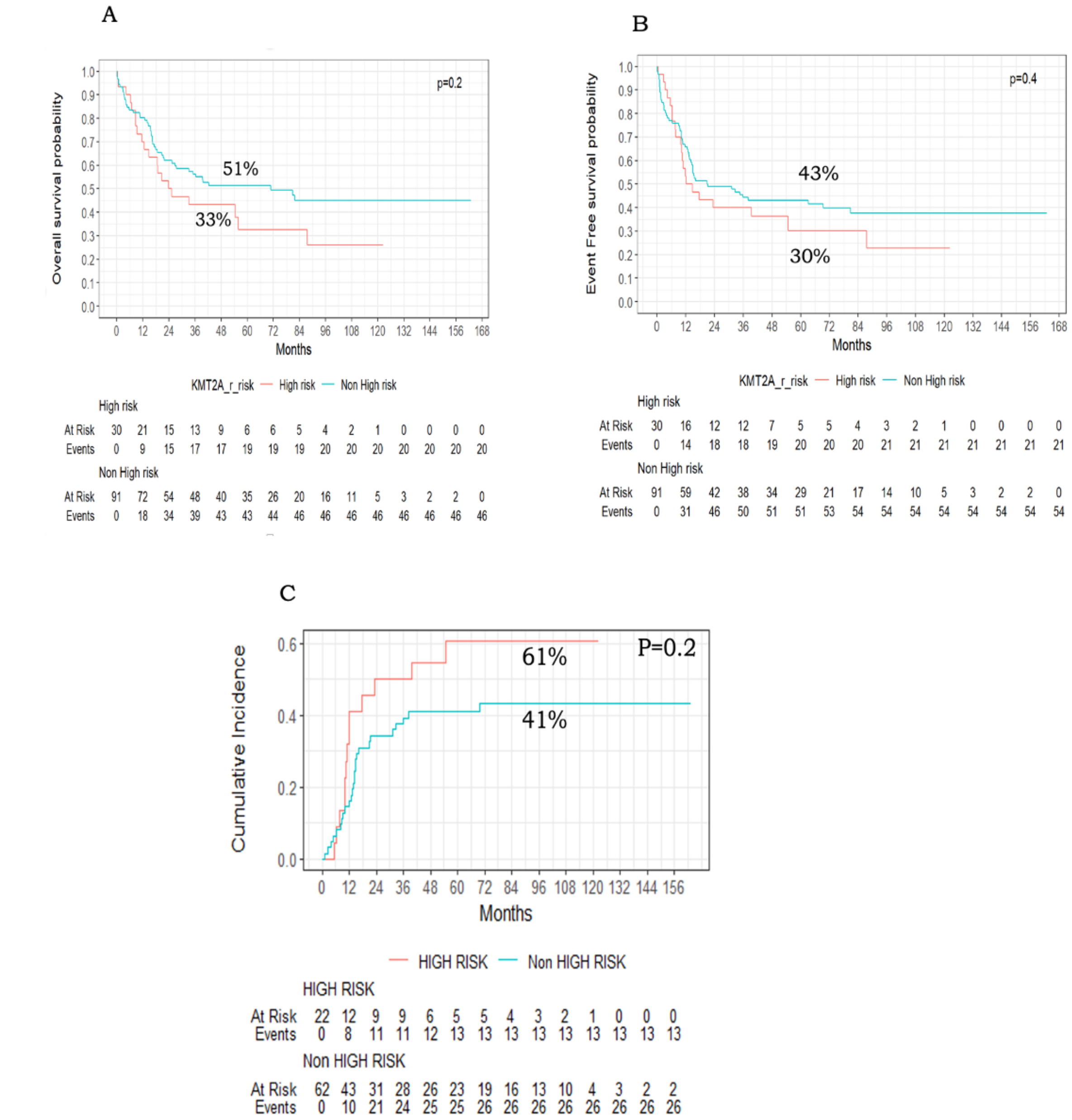

Supplementary Figure 6: Outcomes of KMT2A-r AML Stratified by Fusion-Based Groups into High Risk and Non-High-Risk Groups. (A) OS (Overall survival), **(B)** EFS (Event free survival), **(C)** CIR (Cumulative incidence of relapse)

A

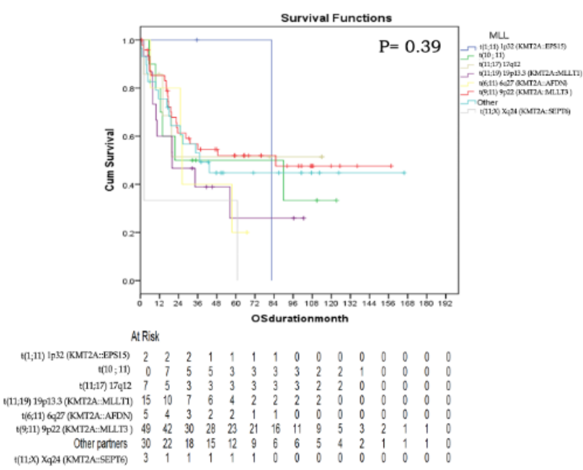

B

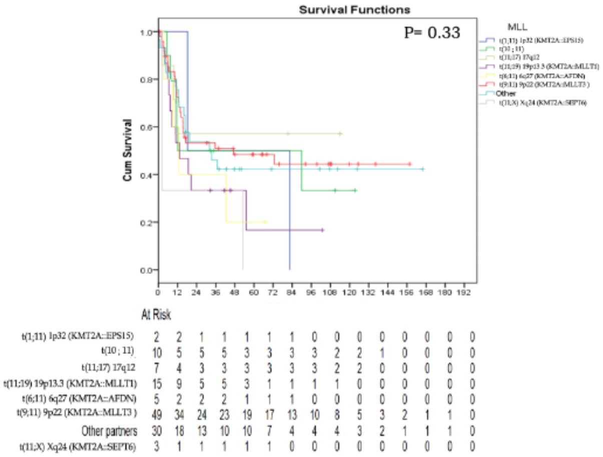

C

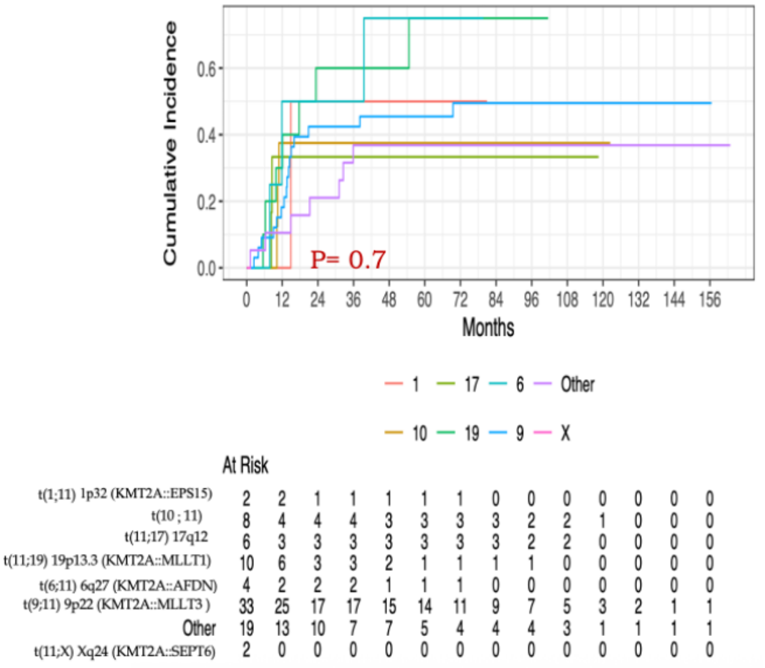

Supplementary Figure 7: Outcomes of KMT2A-r AML fusion partners. (A) OS (Overall survival), **(B)** EFS (Event free survival), **(C)** CIR (Cumulative incidence of relapse)
